# Persistent left superior vena cava is associated with complex atrial tachyarrhythmias in repaired tetralogy of Fallot: evidence for a right-sided arrhythmogenic substrate

**DOI:** 10.1016/j.ijcha.2026.101930

**Published:** 2026-04-20

**Authors:** Tabito Kino, Miyako Igarashi, Yuta Suto, Yasutoshi Shinoda, Naoto Kawamatsu, Kojiro Ogawa, Yuki Komatsu, Tomoko Machino-Ohtsuka, Hiro Yamasaki, Tomoko Ishizu

**Affiliations:** Department of Cardiology, Institute of Medicine, University of Tsukuba. 1-1-1 Tennodai, Tsukuba, Ibaraki 305-8575, Japan

**Keywords:** Atrial fibrillation, Catheter ablation, Repaired tetralogy of Fallot, Persistent left superior vena cava

## Abstract

**Background:**

Atrial fibrillation (AF) is an important late complication in patients with repaired tetralogy of Fallot (TOF); however, its clinical determinants and arrhythmogenic substrates remain uncertain. Identifying high-risk anatomical profiles is essential for optimal rhythm management and tailored ablation strategies.

**Methods:**

We retrospectively analyzed 137 consecutive patients with repaired TOF followed at the University of Tsukuba Hospital (2013–2024). Clinical characteristics, echocardiographic parameters, and procedural findings were compared between patients with and without AF. Logistic regression identified independent predictors. Catheter ablation approaches and outcomes were evaluated in patients who underwent AF ablation.

**Results:**

AF occurred in 14 patients (10.2%), frequently coexisting with atrial tachycardia (78.6%). Compared with patients without AF, those with AF were older, had undergone more repeat cardiac surgeries, and more commonly exhibited a persistent left superior vena cava (PLSVC), greater biatrial enlargement, and mildly reduced right ventricular function. On multivariable analysis, PLSVC remained statistically associated with AF (odds ratio 15.5, p = 0.002); however, this association was primarily driven by patients with combined AF and AT, as no PLSVC was observed in the small isolated AF subgroup. Among six patients who underwent catheter ablation, those without PLSVC were successfully treated with pulmonary vein isolation (PVI) alone, whereas two patients with PLSVC underwent right-sided ablation targeting the right atrium and coronary sinus, with no AF recurrence observed during follow-up.

**Conclusions:**

PLSVC was associated with more complex atrial tachyarrhythmias, particularly in patients with concomitant AF and AT. Recognizing this substrate may inform individualized ablation strategies beyond conventional PVI.

## 1 Introduction

Advancements in surgical strategies and perioperative management have significantly improved the survival rates of patients with tetralogy of Fallot (TOF), allowing most to reach adulthood [1], [2]. Although surgical repair during infancy or childhood effectively alleviates hemodynamic abnormalities, late complications, particularly atrial arrhythmias such as atrial fibrillation (AF), have emerged as significant long-term concerns [3]. AF in patients with repaired TOF is increasingly recognized as a clinically important condition due to its association with heart failure and reduced quality of life [4]. However, AF occurs less frequently than atrial tachycardia (AT), which is common in the late postoperative phase, and the substrate characteristics specific to repaired TOF remain poorly defined.

Persistent left superior vena cava (PLSVC) is a relatively uncommon venous anomaly, occurring in approximately 0.3–0.5% of the general population and in 4–8% of patients with congenital heart disease [5]. Although often considered an incidental anatomical variant, PLSVC has been reported to contain myocardial sleeves and potential ectopic foci that may contribute to atrial arrhythmogenesis. However, its clinical relevance in patients with repaired TOF remains insufficiently characterized.

Although left ventricular (LV) dysfunction, aging, prior cardiac surgeries, and left atrial (LA) enlargement have all been associated with AF in repaired TOF, these features do not fully account for its underlying mechanisms. Prior work has shown that AF becomes more prevalent in individuals aged over 45 years [3].

Catheter ablation is now an established treatment option for AF in the general population; however, in patients with adult congenital heart disease, altered atrial anatomy and extensive atriotomy scars pose significant challenges to procedural success [6]. Data remain limited regarding the optimal ablation strategy in this population, including the roles of pulmonary vein isolation (PVI), linear ablation, and substrate modification. Moreover, few studies have systematically evaluated the procedural characteristics, outcomes, and recurrence patterns of AF ablation in repaired TOF patients.

We aimed to characterize the clinical and electrophysiological features of AF in patients with surgically repaired TOF and to investigate the efficacy of catheter ablation strategies in this population. By identifying arrhythmogenic patterns and ablation outcomes, we seek to inform the development of more effective and tailored therapeutic approaches for this high-risk group.

## 2 Methods

### 2.1 Study design

This retrospective, single-center observational study was conducted from 2013 to 2024 at the University of Tsukuba Hospital to evaluate the clinical characteristics and treatment outcomes of AF. This study was also conducted in accordance with the principles of the Declaration of Helsinki and was approved by the Institutional Review Board of University of Tsukuba Hospital (Approval No. R05-255). Owing to the retrospective nature of the study, the need for written informed consent was waived.

### 2.2 Clinical characteristics

All patients who underwent complete intracardiac repair for TOF were included in this study. Patients with a documented history of AF were identified using electrocardiogram (ECG) data. AF was defined as documented episodes occurring either prior to enrollment or during the follow-up period. Rhythm surveillance was performed as part of routine clinical follow-up and included standard 12-lead ECG at outpatient visits, Holter monitoring when clinically indicated, and interrogation of cardiovascular implantable electronic devices in patients with available devices. Continuous systematic rhythm monitoring was not uniformly applied to all patients because of the retrospective study design. Clinical, electrocardiographic, and transthoracic echocardiographic data were collected at enrollment, and additional information was obtained during the periprocedural and long-term follow-up phases. These parameters were compared between patients with AF and those without AF. In the present study, AT included both focal AT and atrial flutter (typical and atypical). The presence of PLSVC was determined based on contrast-enhanced computed tomography (CT; 12 patients), non-contrast-enhanced cardiac magnetic resonance imaging (two patients), and/or transthoracic echocardiography findings (one patient). When multiple imaging modalities were available, cross-sectional imaging findings were prioritized. One case of PLSVC was identified based on operative or medical record documentation. In all identified cases, the PLSVC drained into the coronary sinus (CS).

### 2.3 Clinical outcomes

The primary clinical outcome was the occurrence or recurrence of AF, documented by ECG, Holter monitoring, or cardiovascular implantable electronic device data during follow-up. Recurrence was defined as any episode of AF or AT following a period of sinus rhythm after intervention or medical management. Secondary outcomes included the need for catheter ablation. All outcome events were independently reviewed and confirmed by at least two electrophysiologists. Follow-up data were collected from medical records, device interrogation reports, and scheduled outpatient visits.

### 2.4 Statistical methods

Continuous variables are expressed as medians with interquartile range (IQR, Q1–Q3), and categorical variables as counts and percentages. Baseline characteristics were compared using the chi-squared or Fisher’s exact test for categorical variables and the Mann–Whitney *U* test or Kruskal–Wallis test for continuous variables. Univariable and multivariable logistic regression analyses were performed to identify predictors of AF. Candidate variables were first screened by univariable logistic regression (p < 0.05) and then assessed for clinical relevance. In total, 12 candidate variables were evaluated. Multicollinearity was assessed using the variance inflation factor (VIF) and pairwise correlation coefficients; no variables met the predefined thresholds for exclusion (VIF ≥ 5 or |r| > 0.7). Given the limited number of AF events, model parsimony was prioritized to avoid overfitting. The final multivariable model was selected using the Bayesian Information Criterion (BIC). Accordingly, AT, β-blocker use, amiodarone use, brain natriuretic peptide, estimated glomerular filtration rate, LA volume index (LAVI), right atrial (RA) area, PR duration, and RA pressure (≥8 mmHg) were not retained in the final multivariable model. Missing data were minimal and handled using complete-case analysis. Odds ratios (ORs) and 95% confidence intervals (CIs) were calculated, and statistical significance was defined as p < 0.05. All statistical analyses were performed using R software, version 4.5.1 (R Foundation for Statistical Computing, Vienna, Austria).

## 3 Results

### 3.1 Distribution of arrhythmias in patients with repaired TOF

**Fig. 1:**
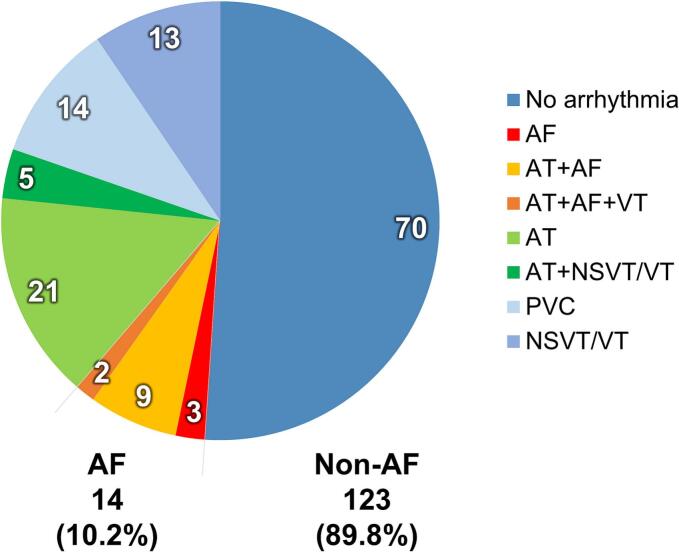
Distribution of arrhythmia types according to atrial fibrillation (AF) presence.

Among 137 patients with repaired TOF, AF was detected in 14 (10.2%) during a median follow-up of 7.0 years (IQR 3.7–10.6 years) (Fig. 1). AF occurred as an isolated arrhythmia in three patients, in combination with AT in nine, and with AT and ventricular tachycardia (VT) in two. Of the remaining 123 patients without AF, 70 had no arrhythmic events, whereas others exhibited AT (n = 21), premature ventricular contraction (PVC; n = 14), non-sustained VT (NSVT)/VT (n = 13), or combined AT and NSVT/VT (n = 5). Only one patient presented with focal AT as the initial arrhythmia, whereas most patients exhibited macro-reentrant or mixed atrial tachyarrhythmias.

### 3.2 Clinical characteristics between AF and non-AF patients

Baseline characteristics of patients with and without AF are summarized in Table 1. Patients with AF were significantly older (49.0 vs. 34.0 years, p < 0.001). The prevalence of PLSVC was markedly higher in the AF group (35.7% vs. 8.9%, p = 0.012), and these patients had undergone more repeat surgeries (≥3: 57.1% vs. 20.3%, p = 0.005). Concomitant AT was also more frequent in the AF group (78.6% vs. 21.1%, p < 0.001). Paroxysmal AF accounted for 42.9% of cases among patients with AF. Echocardiographic findings revealed greater atrial enlargement among AF patients, including LAVI and RA area, with both atria comparably enlarged. Right ventricular fractional area change (RVFAC) was slightly lower in the AF group. No significant intergroup differences were observed in LV function, valvular regurgitation, or electrocardiographic parameters.

**Table 1:** Baseline characteristics of enrolled patients in the atrial fibrillation (AF) and non-AF groups.

	AF (n = 14)	Non-AF (n = 123)	p value
**Age (years)**	**49.0 [37.5–59.5]**	**34.0 [28.0–44.0]**	**<0.001**
Male	10 (71.4)	58 (47.2)	0.098
Body weight (kg)	57.8 [52.4**–**69.2]	56.2 [48.9**–**68.0]	0.717
BMI (kg/m^2^)	23.0 [20.5**–**26.7]	21.7 [19.8**–**25.2]	0.652
Paroxysmal AF	6 (42.9)	–	–
Comorbidities			
Hypertension	0 (0.0)	5 (4.1)	1.000
Diabetes	2 (14.3)	4 (3.3)	0.115
Dyslipidemia	0 (0.0)	5 (4.1)	1.000
Stroke	2 (14.3)	4 (3.3)	0.115
Coronary artery disease	1 (7.1)	0 (0.0)	0.102
Chronic kidney disease	0 (0.0)	2 (1.6)	1.000
Cardiovascular implantable device	4 (28.6)	13 (10.6)	0.075
Concomitant arrhythmias			
**AT**	**11 (78.6)**	**26 (21.1)**	**<0.001**
NSVT	0 (0.0)	11 (8.9)	0.603
VT	2 (14.3)	13 (10.6)	0.652
**Total number of surgeries (≥3)**	**8 (57.1)**	**25 (20.3)**	**0.005**
**PLSVC**	**5 (35.7)**	**11 (8.9)**	**0.012**
Medication			
β**-blocker**	**9 (64.3)**	**31 (25.2)**	**0.004**
**Amiodarone**	**5 (35.7)**	**2 (1.6)**	**<0.001**
Laboratory data			
Hb (g/dL)	13.6 [12.3**–**14.4]	13.7 [12.7**–**14.9]	0.474
AST (U/L)	26.5 [17.8**–**40.0]	20.0 [17.0**–**24.0]	0.081
ALT (U/L)	16.0 [13.3**–**31.0]	16.0 [12.3**–**23.0]	0.456
**eGFR (mL/min)**	**81.4 [67.8–86.5]**	**96.9 [83.9–108.8]**	**0.002**
**BNP (pg/mL)**	**124.2 [64.1–170.0]**	**40.3 [21.7–71.3]**	**<0.001**
Electrocardiogram			
PR interval (ms)	180.0 [158.5**–**241.5]	162.0 [146.5**–**189.5]	0.164
QRS duration (ms)	158.0 [134.0**–**170.0]	138.0 [104.0**–**156.0]	0.061
CRBBB	13 (92.9)	89 (72.4)	0.116
Echocardiography			
LVEF (%)	59.5 [56.0**–**63.0]	60.0 [58.0**–**64.0]	0.409
E/e’	8.2 [7.4**–**9.7]	7.2 [6.0**–**9.4]	0.096
**LAVI (mL/m^2^)**	**39.5 [32.3–46.5]**	**25.0 [21.0–30.5]**	**<0.001**
**RA area (cm^2^)**	**28.9 [22.4–34.4]**	**19.0 [14.6–22.8]**	**<0.001**
RA area/LA area	1.3 [1.0**–**1.6]	1.1 [1.0**–**1.4]	0.315
RAP (≥ 8 mmHg)	6 (42.9)	22 (17.9)	0.074
RVSP (mmHg)	34.0 [29.3**–**55.8]	33.0 [26.0**–**43.8]	0.459
RVEDVI (mL/m^2^)	156.6 [143.8**–**175.5]	152.0 [131.1**–**170.5]	0.377
**RVFAC (%)**	**37.0 [31.3–40.5]**	**40.5 [36.0–46.8]**	**0.032**
S’ (cm/s)	7.6 [6.0**–**10.9]	9.7 [7.7**–**11.8]	0.105
PAV (cm/s)	204.0 [160.5**–**255.8]	208.0 [171.3**–**263.5]	0.816
TR (moderate-severe)	3 (21.4)	11 (8.9)	0.156
PR (moderate-severe)	3 (21.4)	61 (49.6)	0.084

Data are shown as median [IQR, Q1**–**Q3] or n (%). Categorical variables were tested using the chi-squared test or Fisher’s exact test, whereas continuous variables were assessed using the Mann–Whitney *U* test. AT, atrial tachycardia; BNP, brain natriuretic peptide; CRBBB, complete right-bundle branch block; E/e’, ratio of early transmitral flow velocity to early diastolic mitral annular velocity; eGFR, estimated glomerular filtration rate; LAVI, left atrial volume index; LVEF, left ventricular ejection fraction; NSVT, non-sustained ventricular tachycardia; PAV, pulmonary artery velocity; PLSVC, persistent left superior vena cava; PR, pulmonary regurgitation; RAP, right atrial pressure; RVEDVI, right ventricular end-diastolic volume index; RVFAC, right ventricular fractional area change; RVSP, RV systolic pressure; S’, systolic tricuspid annular velocity; TR, tricuspid regurgitation.

### 3.3 Risk factors for AF

Univariable logistic regression identified several factors significantly associated with the development of AF (Table 2). These included older age (OR 1.07, p = 0.001), concomitant AT (OR 13.7, p < 0.001), a greater number of previous cardiac surgeries (≥3; OR 5.23, p = 0.005), and presence of PLSVC (OR 5.66, p = 0.011). In addition, prolonged PR interval, greater LAVI, larger RA area, and elevated RA pressure (RAP ≥ 8 mmHg) were associated with AF in univariable analysis, reflecting atrial remodeling and increased right-sided loading conditions. In multivariable logistic regression analysis, PLSVC remained independently associated with AF (OR 15.5, 95% CI 2.84–84.6, p = 0.002), along with age (OR 1.12, 95% CI 1.05–1.20, p = 0.001) and a history of more than three cardiac surgeries (OR 11.0, 95% CI 2.34–51.8, p = 0.002). These findings indicate that anatomical factors such as PLSVC and atrial remodeling, rather than ventricular dysfunction, play pivotal roles in AF development among patients with repaired TOF. In a supplementary analysis stratified by PLSVC status, atrial tachyarrhythmias (AF and AT) were more frequently observed in patients with PLSVC than in those without PLSVC (Table S1), supporting the concept that PLSVC is associated with a higher burden and complexity of atrial tachyarrhythmias rather than with isolated AF.

**Table 2:** Univariable and multivariable logistic regression evaluating predictors of atrial fibrillation (AF) development.

Factors	Univariable		Multivariable	
	Odds ratio [95% CI]	p value	Odds ratio [95% CI]	p value
**Age (years)**	**1.07 [1.03–1.12]**	**0.001**	**1.12 [1.05–1.20]**	**0.001**
AT	13.7 [3.55–52.7]	<0.001		
**Total number of surgeries (≥3)**	**5.23 [1.66–16.4]**	**0.005**	**11.0 [2.34–51.8]**	**0.002**
**PLSVC**	**5.66 [1.61–19.9]**	**0.011**	**15.5 [2.84–84.6]**	**0.002**
PR interval (ms)	1.01 [1.00–1.02]	0.045		
LAVI (mL/m^2^)	1.05 [1.02–1.09]	0.002		
RA area (cm^2^)	1.15 [1.07–1.23]	<0.001		
RAP (≥ 8 mmHg)	3.37 [1.06–10.7]	0.039		
RVFAC	0.93 [0.88–1.00]	0.063		

Data are shown as odds ratio [95% confidence interval (CI)]. AT, atrial tachycardia; LAVI, left atrial volume index; PLSVC, persistent left superior vena cava; RAP, right atrial pressure; RVFAC, right ventricular fractional area change.

### 3.4 Management and outcomes of AF in patients with repaired TOF

Among the 14 patients with AF, eight had paroxysmal AF. Of the total cohort, eight patients were managed with medication alone, seven with paroxysmal, and one with persistent AF. Paroxysmal AF was mainly treated with β-blockers, whereas persistent AF was managed primarily with amiodarone. One patient with paroxysmal AF could not tolerate either a β-blocker or amiodarone because of bradycardia but was able to maintain sinus rhythm with heart failure medications. Another patient with persistent AF developed worsening heart failure and underwent mitral and tricuspid valvuloplasty as well as pulmonary valve replacement; however, sinus rhythm could not be restored despite continued amiodarone therapy. The remaining six patients (42.9%) underwent catheter ablation, and their procedural characteristics are summarized in Table 3. Of these, four were treated with LA-based strategies (Cases 1–4), including PVI with or without additional LA defragmentation, whereas two patients with concomitant PLSVC (Cases 5–6) underwent exclusively right-sided ablation targeting the RA and CS. Recurrence occurred in two patients. One (Case 2) had PV reconnection and underwent repeat isolation. The other (Case 5) developed recurrent AT. This AT was caused by a reentrant circuit involving the CS ostium, which had undergone defragmentation during the previous procedure. Linear ablation was performed across the CS ostium roof between the pre-existing scars, successfully terminating the AT. Although AF did not recur, PVI was also performed during this session. Antiarrhythmic drug therapy, predominantly β-blockers and amiodarone, was frequently prescribed in conjunction with catheter ablation. These findings highlight the heterogeneity of ablation strategies in repaired TOF and the unique influence of PLSVC on procedural decision-making.

**Table 3:** Summary of atrial fibrillation (AF) ablation cases (Cases 1–6) in patients with repaired tetralogy of Fallot.

Case	Age	Gender	Type of AF	PLSVC	1st session		Recurrence	2nd session	Medication
					PVI	Other			
1	64	Male	Paroxysmal	–	+	–	–	NA	β-blocker
2	48	Female	Persistent	–	+	–	AF	PV re-isolation	β-blocker
3	65	Male	Persistent	–	+	–	–	NA	β-blocker
4	37	Male	Persistent	–	+	LA defragmentation	–	NA	Amiodarone
5	44	Male	Persistent	**+**	–	RA/CS defragmentation	AT	CS ostium linear ablation for reentrant AT, PVI	β-blocker, Amiodarone
6	33	Male	Persistent	**+**	–	RA/CS defragmentation	–	NA	β-blocker, Amiodarone

AT, atrial tachycardia; CS, coronary sinus; NA, not applicable; PLSVC, persistent left superior vena cava; PVI, pulmonary vein isolation.

**Fig. 2:**
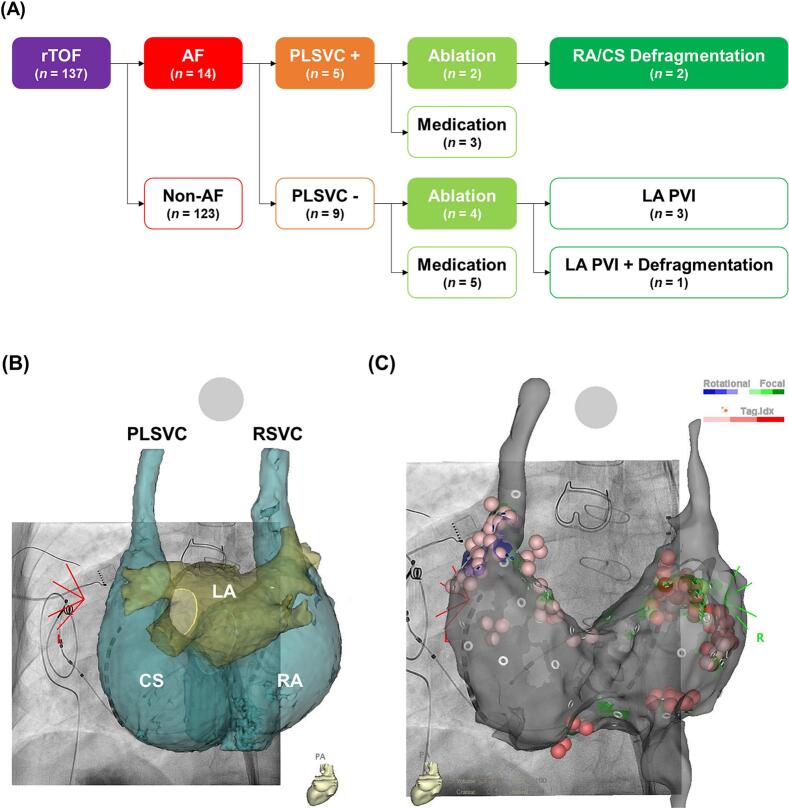
Patient stratification and ablation strategy in repaired Tetralogy of Fallot (TOF) patients with and without persistent left superior vena cava (PLSVC). (A) Flowchart of patient selection and comparison based on the presence of PLSVC. (B) Merged contrast-enhanced CT and 3D electroanatomical mapping demonstrate a markedly dilated RA and CS associated with the presence of a PLSVC, visualized using the CARTO3 system (Biosense Webster, Irvine, CA). (C) Detailed ablation sites (red tags) of RA and CS defragmentation are displayed on the anatomical map. CS, coronary sinus; RA, right atrium; RSVC, right superior vena cava; TV, tricuspid valve; MV, mitral valve. (For interpretation of the references to colour in this figure legend, the reader is referred to the web version of this article.)

To further investigate the potential arrhythmogenic role of PLSVC, clinical and procedural findings were compared between AF patients with and without PLSVC (Fig. 2A). Patients with PLSVC more frequently had persistent AF (60% vs. 33%). In these patients, ablation lesions were localized mainly on the right side, targeting RA and CS defragmentation, without any LA intervention, including PVI. Integration of contrast-enhanced CT with 3D electroanatomical mapping revealed a PLSVC with marked RA and CS dilatation (Fig. 2B). Using the CARTO3^TM^ system (Biosense Webster, Irvine, CA, USA), anatomical abnormalities contributing to arrhythmogenesis were identified. Guided by CARTO FINDER^TM^ (Biosense Webster), ablation sites were concentrated in the RA and CS regions for defragmentation in two representative cases (Case 5 and 6) (Fig. 2C) [7]. In the phenotype-stratified analysis, PLSVC was more prevalent in the mixed AT/AF group (5/11, 45.5%) than in the pure AT group (3/26, 11.5%) and the group without atrial tachyarrhythmia (8/97, 8.2%). No PLSVC was observed in the small pure AF subgroup (0/3) (Table S2). This distribution suggests that PLSVC may be associated with arrhythmia complexity rather than isolated AF.

## 4 Discussion

### 4.1 Risk factors for AF in patients with repaired TOF

In this retrospective study of patients with repaired TOF, AF was detected in approximately 10% of patients, a prevalence consistent with previous reports (7.4–21%) [3], [8]. AF frequently coexisted with AT but occurred at a markedly lower frequency compared with AT (30%) [9]. AF was associated with distinct clinical and structural characteristics, including older age, greater atrial enlargement, mildly reduced RV systolic function, and the presence of a PLSVC (Table 1). Previously reported risk factors for AF in patients with repaired TOF include advanced age, prior cardiac surgeries, LA dilatation, and LV dysfunction, which are primarily associated with the left heart [2], [3]. In contrast, risk factors for AT, such as prior cardiac surgeries, hypertension, and RA dilatation, are predominantly right heart [2], [3], [9]. In our cohort, overlap between AF and AT was common, likely reflecting shared atrial remodeling processes or the possibility of AT acting as a trigger for AF. The diagnostic boundary between AF and AT in congenital heart disease can be ambiguous, especially in the presence of complex atrial substrates and multiple arrhythmogenic mechanisms. In this study, atrial flutter was included within the AT category; therefore, the observed overlap between AF and organized atrial tachyarrhythmias likely reflects shared right-atrial substrate in repaired TOF. We acknowledge that these arrhythmias have distinct electrophysiological mechanisms; however, in patients with repaired TOF, multiple arrhythmia types frequently coexist and evolve over time, making strict categorization challenging.

### 4.2 Association between PLSVC and AF development

A key finding of this study was that PLSVC was associated with AF occurrence; however, this relationship appears to reflect more complex atrial tachyarrhythmias, primarily driven by patients with combined AF and AT rather than isolated AF. However, the phenotype-stratified analysis revealed that PLSVC was most enriched in patients with concomitant AF and AT rather than in those with isolated AF (Table 1). Multivariable analysis confirmed its strong association with AF (OR 15.5, p = 0.002; Table 2), with an effect size exceeding that of conventional remodeling markers such as age and LAVI. This suggests that PLSVC may represent a distinct arrhythmogenic substrate rather than a secondary manifestation of atrial remodeling or hemodynamic load. Although PLSVC is rare in the general population (0.3–0.5%), its prevalence is notably higher in patients with congenital heart disease (4–8%) [10]. Its independent association with AF in repaired TOF highlights its clinical relevance. These findings suggest that PLSVC should not be regarded as an incidental anatomical variant but rather as a clinically relevant marker of a complex right-sided arrhythmogenic substrate in this population. Anatomically, PLSVC may contain residual pacemaker cells and myocardial sleeves capable of generating ectopic electrical activity [11], [12], whereas concomitant CS dilatation and RA remodeling can facilitate reentrant or focal atrial arrhythmias [13], [14]. In the present study, although only two patients with PLSVC-related AF underwent right-sided ablation, RA/CS defragmentation without PVI effectively suppressed AF in both cases. This finding suggests that AF in patients with repaired TOF and PLSVC may not follow the typical LA pattern seen in the general population but may instead originate from right-sided substrates involving the RA, CS, or PLSVC. Consequently, in this subset of patients, substrate modification targeting these right-sided structures, rather than conventional PVI alone, may be required to achieve durable rhythm control [15]. Therefore, the association between PLSVC and AF should be interpreted as hypothesis-generating, particularly given the small number of isolated AF cases. Importantly, this association was largely driven by patients with combined AF and AT, rather than isolated AF. In our cohort, only one patient presented with focal AT as the initial arrhythmia, suggesting that focal AT rarely exists as an isolated phenotype in this population. These findings indicate that PLSVC may represent a right-sided arrhythmogenic substrate associated with arrhythmia progression and complexity, rather than a specific determinant of isolated AF.

### 4.3 Atrial remodeling, right-sided hemodynamic burden, and synergistic effects with PLSVC

Beyond the structural contribution of PLSVC, our findings highlight the importance of right-sided hemodynamic burden as an additional determinant of AF in repaired TOF. Reduced RVFAC, prolonged PR interval, and elevated RAP were identified as risk factors for AF development (Table 2). These indices reflect chronic RV volume and pressure overload leading to progressive RA dilatation, increased wall stress, and conduction heterogeneity. Such remodeling not only promotes AF independently but may also amplify PLSVC-related arrhythmogenicity through enhanced electrical coupling and increased atrial stretch [1], [2], [3], [8]. Collectively, these findings suggest that structural variations such as PLSVC and functional loading conditions act synergistically to create a complex biatrial arrhythmogenic substrate in repaired TOF. Accordingly, optimizing RA and RV loading conditions, alongside tailored substrate modification targeting the RA–CS–PLSVC continuum, may be crucial for durable rhythm control.

### 4.4 Implications for ablation strategy

These mechanistic insights have direct implications for the interventional management of AF in repaired TOF. In our cohort, the presence of PLSVC influenced ablation strategy, with lesions localized mainly to the right side, targeting RA and CS defragmentation without transseptal LA access or PVI. This tailored approach, guided by contrast-enhanced CT and 3D electroanatomical mapping, corresponded to the anatomical distribution of the arrhythmogenic substrate (Fig. 2). Such a right-sided, substrate-guided strategy contrasts with the standard PVI-focused approach used in the general AF population, where the LA is typically the primary therapeutic target [15], [16].

In patients with complex congenital anatomy, particularly those with venous anomalies or extensive surgical scarring, conventional strategies may be suboptimal. Instead, preprocedural imaging combined with individualized, substrate-guided ablation may improve efficacy and safety [17], [18]. Collectively, these results suggest that in patients with repaired TOF, especially those with PLSVC and right-sided hemodynamic burden, the arrhythmogenic substrates may predominantly reside in the RA-CS-PLSVC complex, and right-sided modification, rather than conventional PVI, may be essential for durable rhythm control. Because PLSVC may be associated with greater congenital complexity and surgical burden, residual confounding cannot be completely excluded despite multivariable adjustment. We also cannot rule out confounding related to underlying congenital anatomy or cumulative surgical burden, although we adjusted for repeat cardiac surgeries as a surrogate marker of surgical complexity. Therefore, PLSVC may partly represent a marker of a more complex atrial substrate.

### 4.5 Limitations

Some limitations should be acknowledged. First, this was a retrospective, single-center study, which may limit generalizability and introduce referral bias. Second, the number of patients with AF, particularly those undergoing catheter ablation, was relatively small, reflecting the inherently low prevalence of AF in repaired TOF compared with AT and the highly selected nature of patients referred for invasive rhythm control. Third, ablation strategies were not standardized and were determined based on individual anatomical and clinical considerations, precluding direct comparison between right-sided and conventional LA approaches. Fourth, detailed electroanatomical mapping data regarding AF initiation and maintenance were not uniformly available, and causal relationships between PLSVC and AF cannot be definitively established. In addition, long-term follow-up data regarding arrhythmia recurrence and procedural outcomes were limited. Fifth, rhythm monitoring was not uniform across the cohort, and differences in surveillance intensity might have affected the detection of asymptomatic or paroxysmal AF. Furthermore, the relatively small number of AF events resulted in a low events-per-variable ratio, which might have increased the risk of model overfitting despite the use of the BIC for model parsimony. Therefore, our findings should be interpreted as hypothesis-generating. Finally, the presence of an innominate vein bridge was not systematically assessed. In addition, the classification of atrial tachyarrhythmias was limited by the coexistence of multiple mechanisms within individual patients, and strict separation of focal AT, atrial flutter, and macro-reentrant AT was not always feasible.

Despite these limitations, the observed association between PLSVC and AF was consistent across the entire repaired TOF cohort and demonstrated a robust effect size exceeding that of conventional atrial remodeling markers. These findings suggest that PLSVC represents a clinically meaningful anatomical substrate rather than a coincidental venous variant and provide a strong rationale for future multicenter, prospective studies incorporating systematic mapping and standardized ablation protocols.

## 5 Conclusions

In this retrospective study of patients with repaired TOF, AF was observed in approximately 10% of cases. Although several indicators of atrial remodeling were associated with AF, PLSVC was associated with complex atrial tachyarrhythmias, and this relationship should be interpreted as hypothesis-generating. These findings suggest that PLSVC represents a clinically relevant right-sided arrhythmogenic substrate rather than merely a coincidental venous anomaly. Recognition of PLSVC may be important for risk stratification and procedural planning, and right-sided, substrate-guided ablation strategies warrant further investigation in this population.

## CRediT authorship contribution statement

**Tabito Kino:** Writing – original draft, Visualization, Validation, Investigation, Formal analysis, Data curation. **Miyako Igarashi:** Writing – review & editing, Project administration, Methodology, Conceptualization. **Yuta Suto:** Writing – original draft, Validation, Investigation, Formal analysis, Data curation. **Yasutoshi Shinoda:** Data curation. **Naoto Kawamatsu:** Resources, Data curation. **Kojiro Ogawa:** Data curation. **Yuki Komatsu:** Writing – review & editing. **Tomoko Machino-Ohtsuka:** Resources, Project administration. **Hiro Yamasaki:** Writing – review & editing. **Tomoko Ishizu:** Writing – review & editing, Supervision.

## Declaration of competing interest

The authors declare the following financial interests/personal relationships which may be considered as potential competing interests: Dr. Igarashi received endowments from Japan Lifeline, Astec Co. Ltd., Medtronic, DVX, Boston Scientific, Abbott Medical Inc., and Biotronik. Dr. Komatsu received honoraria for lectures from Biosense Webster. The remaining authors have no conflicts of interest to disclose.
