# Supplementary material for: Persistent left superior vena cava is associated with complex atrial tachyarrhythmias in repaired tetralogy of Fallot: evidence for a right-sided arrhythmogenic substrate
Source: Int J Cardiol Heart Vasc. 2026 Apr 20;64:101930. doi: 10.1016/j.ijcha.2026.101930 (PMC13122695; doi:10.1016/j.ijcha.2026.101930)
Supplement: Supplementary Data 1 [file mmc1.docx]

**Supplementary Data**

**Persistent Left Superior Vena Cava is Associated with Complex Atrial Tachyarrhythmias, Including Atrial Fibrillation, in Repaired Tetralogy of Fallot: Evidence for a Right-Sided Arrhythmogenic Substrate**

Tabito Kino, Miyako Igarashi, Yuta Suto, Yasutoshi Shinoda, Naoto Kawamatsu, Kojiro Ogawa, Yuki Komatsu, Tomoko Machino-Ohtsuka, Hiro Yamasaki, Tomoko Ishizu

Department of Cardiology, Institute of Medicine, University of Tsukuba.

**Supplementary Tables**

**Table S1.** Phenotype distribution of atrial tachyarrhythmias in patients with and without PLSVC.

**Table S2.** Phenotype-stratified analysis of atrial tachyarrhythmias.

**Table S1.** Phenotype distribution of atrial tachyarrhythmias in patients with and without PLSVC.

|  | PLSVC (-)  (n = 121) | PLSVC (+)  (n = 16) | p value |
| --- | --- | --- | --- |
| AF | 3 (2.5) | 0 (0.0) | 0.012 |
| AT/AF | 6 (5.0) | 5 (31.2) |  |
| AT | 23 (19.0) | 3 (18.8) |  |
| Non-AT/AF | 89 (73.5) | 8 (50.0) |  |

Data are shown as n (%). Categorical variables were tested using the chi-squared test or Fisher’s exact test. The p value refers to the comparison of phenotype distribution between patients with and without PLSVC. AF, atrial fibrillation; AT, atrial tachycardia; PLSVC, persistent left superior vena cava.

**Table S2.** Phenotype-stratified analysis of atrial tachyarrhythmias.

|  | AF  (n = 3) | AT/AF  (n = 11) | AT  (n = 26) | Non-AT/AF  (n = 97) | p value |
| --- | --- | --- | --- | --- | --- |
| **Age (years)** | **57.0 [48.0–59.5]** | **49.0 [41.5–59.5]** | **41.5 [35.0–51.0]** | **34.0 [28.0–42.0]** | **0.001** |
| Male | 2 (66.7) | 8 (72.7) | 12 (46.2) | 46 (47.4) | 0.416 |
| **Total number of surgeries (≥3)** | **2 (66.7)** | **6 (54.5)** | **13 (50.0)** | **12 (12.4)** | **<0.001** |
| **PLSVC** | **0 (0.0)** | **5 (45.5)** | **3 (11.5)** | **8 (8.2)** | **0.012** |
| Hb | 14.8 [14.2–15.5] | 13.1 [11.7–14.0] | 13.5 [11.9–14.5] | 13.8 [12.8–15.0] | 0.150 |
| **BNP (pg/mL)** | **124.2 [86.4–200.2]** | **104.6 [68.2–162.0]** | **57.7 [22.5–111.8]** | **37.9 [21.0–63.1]** | **0.001** |
| LVEF (%) | 63.0 [57.5–63.0] | 59.0 [56.0–62.5] | 61.0 [56.8–63.0] | 60.0 [58.0–64.0] | 0.849 |
| **LAVI (mL/m^2^)** | **45.0 [42.5–54.5]** | **37.0 [29.5–45.0]** | **29.5 [24.3–36.8]** | **24.0 [21.0–29.0]** | **<0.001** |
| **RA area (cm^2^)** | **23.4 [22.7–25.5]** | **32.0 [23.9–35.6]** | **24.8 [21.4–27.6]** | **18.0 [14.3–20.5]** | **<0.001** |
| **RA area /**  **LA area** | **1.1 [0.9–1.1]** | **1.4 [1.0–1.7]** | **1.4 [1.2–1.6]** | **1.1 [0.9–1.2]** | **<0.001** |
| RAP (≥ 8 mmHg) | 1 (33.3) | 5 (18.2) | 6 (15.4) | 16 (7.2) | 0.108 |
| RVFAC (%) | 32.0 [28.5–34.5] | 38.0 [32.0–41.0] | 39.5 [35.3–44.8] | 41.0 [36.0–47.0] | 0.097 |

Data are shown as median [IQR, Q1**–**Q3] or n (%). Categorical variables were tested using the chi-squared test or Fisher’s exact test, whereas continuous variables were assessed using the Kruskal–Wallis test. AF, atrial fibrillation; AT, atrial tachycardia; BNP, brain natriuretic peptide; LAVI, left atrial volume index; LVEF, left ventricular ejection fraction; PLSVC, persistent left superior vena cava; RAP, right atrial pressure; RVFAC, right ventricular fractional area change.
